# Development and operation of a digital platform for sharing pathology image data

**DOI:** 10.1186/s12911-021-01466-1

**Published:** 2021-04-03

**Authors:** Yunsook Kang, Yoo Jung Kim, Seongkeun Park, Gun Ro, Choyeon Hong, Hyungjoon Jang, Sungduk Cho, Won Jae Hong, Dong Un Kang, Jonghoon Chun, Kyoungbun Lee, Gyeong Hoon Kang, Kyoung Chul Moon, Gheeyoung Choe, Kyu Sang Lee, Jeong Hwan Park, Won-Ki Jeong, Se Young Chun, Peom Park, Jinwook Choi

**Affiliations:** 1 Department of Biomedical Engineering, Seoul National University Hospital, Seoul, Republic of Korea; 2 Department of Pathology, Seoul National University Hospital, Seoul, Republic of Korea; 3 Department of Biomedical Engineering, College of Medicine, Seoul National University, Seoul, Republic of Korea; 4 Prompt Technology, Co., Ltd., Seoul, Republic of Korea; 5 Department of Computer Science, Ulsan National Institute of Science and Technology, Ulsan, Republic of Korea; 6 Department of Computer Science and Engineering, Korea University, Seoul, Republic of Korea; 7 Department of Electrical Engineering, Ulsan National Institute of Science and Technology, Ulsan, Republic of Korea; 8 Department of Data Technology, School of Software Convergence, College of ICT Convergence, Myongji University, Seoul, Republic of Korea; 9 Department of Pathology, Seoul National University College of Medicine, Seoul, Republic of Korea; 10 Department of Pathology, Seoul National University Bundang Hospital, Seoul National University College of Medicine, Seongnam, Republic of Korea; 11 Department of Pathology, Seoul National University Bundang Hospital, Seongnam, Republic of Korea; 12 Department of Pathology, Seoul Metropolitan Government-Seoul National University Boramae Medical Center, Seoul National University College of Medicine, Seoul, Republic of Korea; 13 Department of Electrical and Computer Engineering, INMC, Seoul National University, Seoul, Republic of Korea; 14 Department of Industrial Engineering, Ajou University, Suwon, Republic of Korea; 15 Institute of Medical and Biological Engineering, Medical Research Center, Seoul National University, Seoul, Republic of Korea

**Keywords:** Digital pathology, Open platform, Artificial intelligence-assisted annotation, Medical image dataset

## Abstract

**Background:**

Artificial intelligence (AI) research is highly dependent on the nature of the data available. With the steady increase of AI applications in the medical field, the demand for quality medical data is increasing significantly. We here describe the development of a platform for providing and sharing digital pathology data to AI researchers, and highlight challenges to overcome in operating a sustainable platform in conjunction with pathologists.

**Methods:**

Over 3000 pathological slides from five organs (liver, colon, prostate, pancreas and biliary tract, and kidney) in histologically confirmed tumor cases by pathology departments at three hospitals were selected for the dataset. After digitalizing the slides, tumor areas were annotated and overlaid onto the images by pathologists as the ground truth for AI training. To reduce the pathologists’ workload, AI-assisted annotation was established in collaboration with university AI teams.

**Results:**

A web-based data sharing platform was developed to share massive pathological image data in 2019. This platform includes 3100 images, and 5 pre-processing algorithms for AI researchers to easily load images into their learning models.

**Discussion:**

Due to different regulations among countries for privacy protection, when releasing internationally shared learning platforms, it is considered to be most prudent to obtain consent from patients during data acquisition.

**Conclusions:**

Despite limitations encountered during platform development and model training, the present medical image sharing platform can steadily fulfill the high demand of AI developers for quality data. This study is expected to help other researchers intending to generate similar platforms that are more effective and accessible in the future.

## Background

Artificial intelligence (AI) is considered to offer a collective intelligence method to solve problems based on data that is applicable to all research fields or industries. To cope with the COVID-19 pandemic, occurring in 2020, governments, public institutions, and private companies in numerous countries including the United States and South Korea, have been encouraging organizations and individual researchers to make various attempts at improving data sharing, including tracking the patients’ routes, and for developing therapeutics [1–4].

In particular, recent medical imaging studies have actively used AI approaches [5]. Harvard University reported that the use of AI may potentially reduce the error rate of a pathologists’ prediction from 3.4% to 0.5% when a deep-learning system is combined with pathologist predictions, Wang et al. [6] In applying a deep-learning algorithm among patients in the emergency department of Seoul National University Hospital validated the performance of AI approaches in the clinical field with respect to improving the diagnostic accuracy and the waiting time [7].

AI requires large amounts of data, especially annotated data, to yield acceptable outcomes [5, 8–10]. Therefore, large-scale information technology companies, including Amazon Web Services (AWS), Microsoft, and Google have recently released public COVID-19-related datasets such as the “AWS COVID-19 data lake” and “COVID-19 Open Research Dataset”[11–13]. To promote advancements in AI approaches, it is necessary to collect curated and annotated data as learning material; however, data collection for AI training is not a simple task, especially for medical imaging.

The reasons for this challenges are as follows. First, medical images can be of a relatively large size. For example, the size of digital pathological images from a tissue specimen is in the gigapixel range, and contains over a million cells, nuclei, and other cellular structures [14–17]. Considering the characteristics of AI approaches, large amounts of data are required; therefore, the entire training dataset would increase in size [8, 9, 15–18]. Second, annotation of collected medical images is labor-intensive, time-consuming, and expensive because experts have to annotate them manually [8, 15, 17–19]. Finally, medical images contain a personal information; therefore, it is not easy to secure and share medical images while conforming with privacy regulations [8–10].

To address these limitations, the Ministry of Health and Welfare of the Republic of Korea has supported studies to develop and share AI learning materials since 2018; we are currently participating in the first phase (2018–2020) of these studies, and have been carrying them out successfully by constructing a data sharing platform, termed the Pathology Artificial Intelligence Platform (PAIP). Over 1700 digital pathological images have been provided as the AI learning dataset on the PAIP to date, and every image includes a high-quality annotation of the tumor area implemented by expert pathologists. Moreover, we organized a pathology AI challenge as a part of the MICCAI 2019 Grand Challenge [20], which has inspired other researchers to conduct subsequent related studies [21, 22]. Before proceeding to the second phase of the project, we here provide a retrospective review on our experiences and challenges faced during data collection, describe the construction of the platform over the past 2 years, and share the implications of our previous studies testing this platform. This experience can provide guidance for future developments to improve the current platform and develop similar AI-based learning materials.

## Construction and content

### Data collection

Pathological slides for PAIP were selected from resected tissue and biopsy tissue slides acquired by the Department of Pathology at Seoul National University Hospital, Seoul National University Bundang Hospital, and SMG-SNU Boramae Medical Center from 2005 to June 2018. To obtain high-quality data in accordance with ethical standards, the following main principles were premised from the selection stage. First, we targeted histologically confirmed tumor cases in five organs (the liver, colon, prostate, pancreas and biliary tract, and kidney). Pathologists personally selected all of the slides, including at least 30% of tumor and normal tissues. Second, for cases of human-derived materials obtained after February 2013, the patients’ consent to donate specimens was required in accordance with the Enforcement Rule of the Bioethics and Safety Act [23]. Third, cases of dead patients were also included. To confirm the patient’s deaths, we only used the patient’s resident registration number, which was not collected by the researchers directly. Patient deaths were also confirmed through a third party, the Bureau of Medical Information Protection at Seoul National University Hospital. Cases with insufficient clinical or pathological information required for AI learning and cases without donation consent since February 2013, were excluded from the data.

Carcinoma samples of the five major organs were selected among numerous target organs, as their diagnosis rate has displayed an increasing tendency in Korea. Furthermore, some morphologically similar carcinomas were selected from other organs, considering metastases. For histologically identical carcinomas, images were selected with maximally different morphologies. These selection criteria would help AI models to learn from our data and thus expand their applications for diagnosing cancers originating from other organs. The selected histological types of carcinomas were adenocarcinoma, hepatocellular carcinoma, and renal cell carcinoma. Of the total 3100 slides, 400–900 slides from each organ, were finally selected for the AI learning materials (Table 1).

**Table 1:** Number of images acquired by cancer type

Organs	Histology	Number of images
Liver	Hepatocellular carcinoma	600
Colon	Adenocarcinoma	900
Prostate	Adenocarcinoma	600
Pancreas and biliary tract	Adenocarcinoma	600
Kidney	Renal cell carcinoma	400
Total		3100

The selected slides were stained using hematoxylin and eosin or through immunohistochemistry (IHC), and a high-quality slide scanner (Leica Aperio AT2) was used to produce a 20 × whole slide image (WSI) in the SVS file format. The scanner can simultaneously process up to 400 slides, and the time required depends on the size of the sample tissue. The process took approximately 8–10 h in each case. All WSIs generated were stored in a large-capacity server after de-identifying the patient data.

### De-identification

**Fig. 1:**
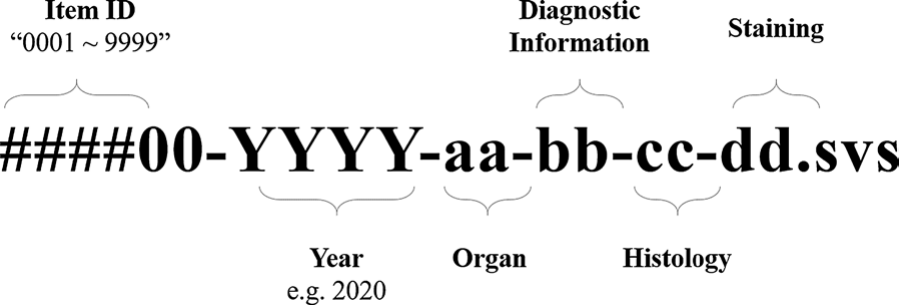
File nomenclature for whole slide image files

The curated data were anonymized at the Department of Pathology, Seoul National University Hospital, except for the information disclosed on the data-sharing platform (organ, histological diagnosis, differentiation, and pathological information). The pathological label assigned to every slide, and the micro-image including that label, were deleted from the WSI file, and the file name was replaced with a temporary research number. Finally, when transferring the data to the learning platform server, the research number was deleted, and the file name of the WSI was randomly assigned in accordance with the file nomenclature (Fig. 1) based on the Guidelines for De-identification of Personal Data of the Republic of Korea (Ministry of Interior and related ministries, 2016) [24]. Several precautions were followed to ensure that any information potentially identifying subjects, including the patients, was not contained in the collected dataset.

### Annotation

De-identified WSIs were directly annotated by one of six pathologists from Seoul National University Hospital, Seoul National University Bundang Hospital, and SMG-SNU Boramae Medical Center. One pathologist from Boramae Hospital annotated 400 kidney cancer images, while the annotation of 900 colon cancer cases was carried out by three pathologists from Seoul National University Hospital. The pathologists used an annotation tool (Aperio) to mark tumor areas and diagnostic information in an XML file format. Thereafter, other pathologists implemented a crosscheck to secure the reliability of the annotation. These annotations were of sufficiently high intensity to complete the process for approximately one slide per day on average when the pathologists performed their clinical tasks in parallel, and it is worth noting that the annotated slide images were considered to provide very valuable data.

**Fig. 2:**
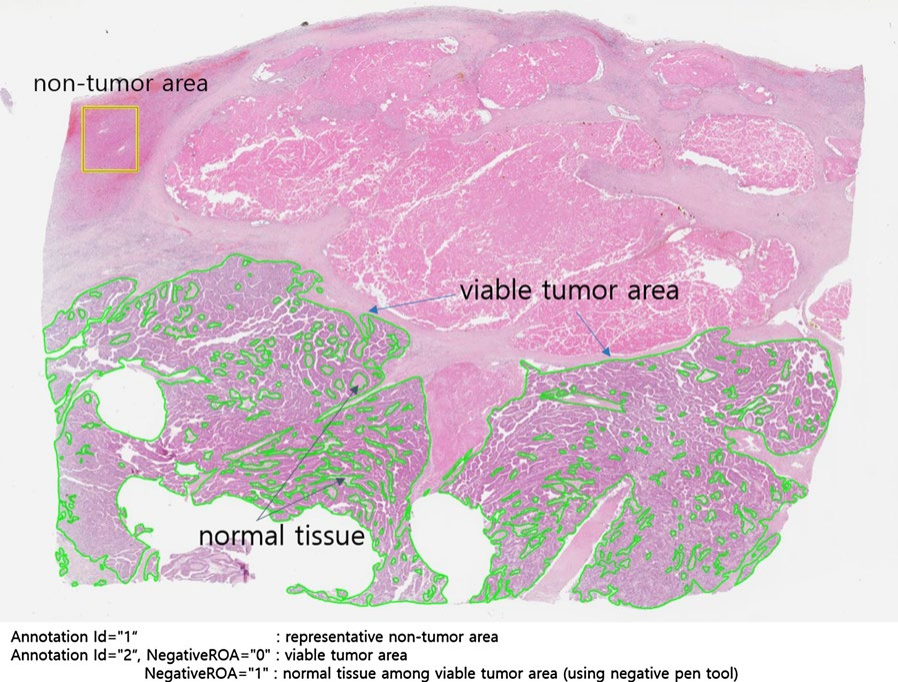
Annotated whole slide image sample

A sample of an annotated WSI is shown in Fig. 2. All WSIs were matched with SVS and XML files at 1:1 pairing, and the annotation layers in the XML file of each cancer type were standardized, helping developers to identify and train medical images upon first handling. An example of tumor annotation in an XML file is as follows. First, the closed boundary was marked in units of the variable tumor. Second, the normal area inside the viable tumor was separated using the negative pen tool (NegativeROA = “1”). Furthermore, typical normal cells outside the viable tumor were marked as the non-tumor area, which was designed to provide developers with abundant training material. The XML thus generated could be overlaid on the SVS file through the viewer to intuitively assess the tumor area, as shown in Fig. 2.

**Fig. 3:**
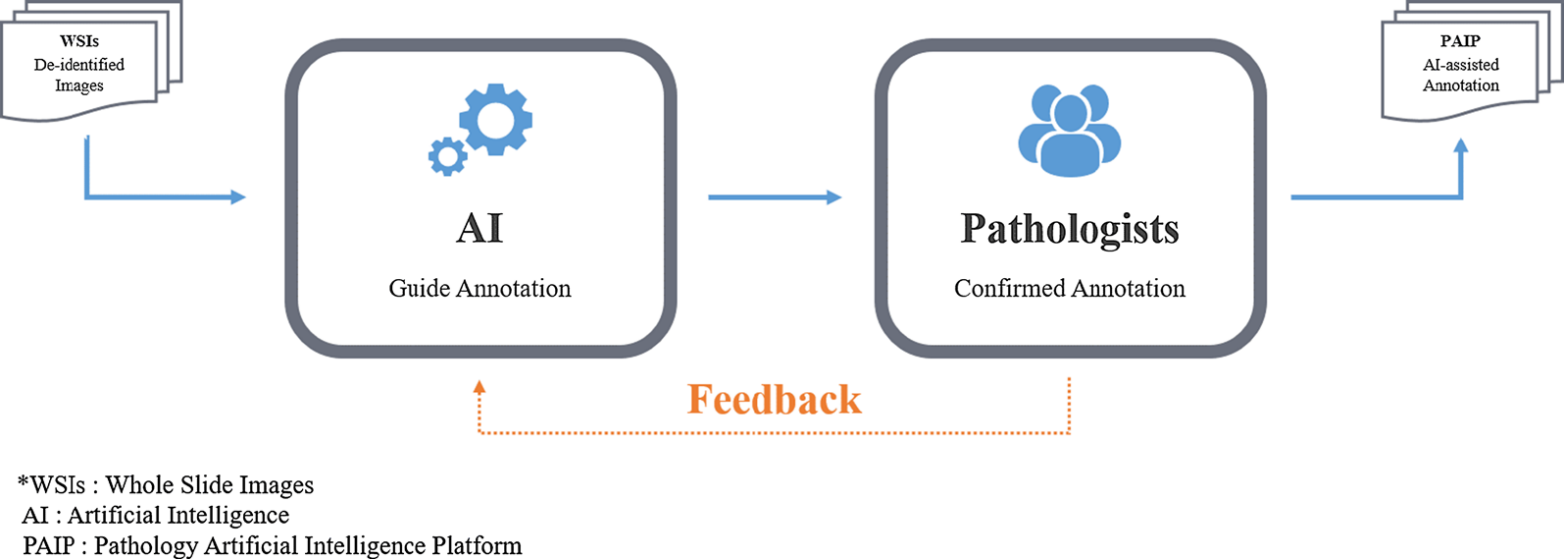
Artificial intelligence-assisted annotation process

**Fig. 4:**
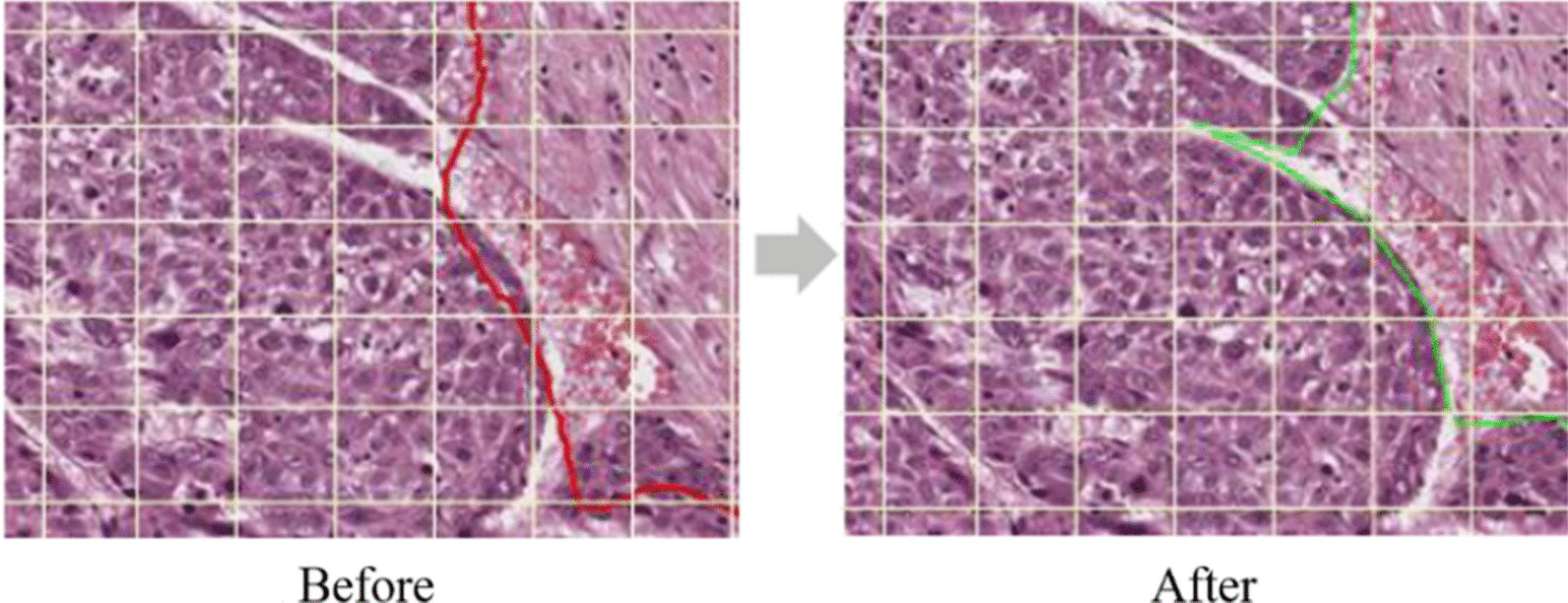
Enhancement by artificial intelligence-assisted annotation process

However, after manual annotation of 900 cases of colorectal cancer, manual slide annotation became challenging owing to the complexity of the boundary of the lesions and the large amount of data required for AI learning. Accordingly, as of 2019, an AI-assisted annotation process was newly established in collaboration with Korea University and Seoul National University AI team (Fig. 3). During the AI-assisted annotation process, annotation was first guided by AI, and the pathologist subsequently examined and corrected the results to confirm the final annotation. This process improved the workload, along with the productivity of the pathologist and the annotation accuracy because it was advantageous for precisely marking the lesion boundary (Fig. 4). The process was first applied for annotating 300 hepatocellular carcinomas after verifying its validity with the PAIP2019 challenge dataset, and was then continuously applied for annotating other organs. Gradually, few manual annotations were able to gather the targeted large size of the dataset (Table 2).

**Table 2:** Status of manual annotation

Organ	Cases	Manual annotation (rate)	AI-assisted annotation (rate)
Colon	900	900 (100%)	0 (−)
Liver	600	300 (50%)	300 (50%)
Prostate	600	310 (52%)	290 (48%)
Kidney	400	103 (26%)	297 (74%)
Pancreas and biliary track	600	10 (2%)	590 (98%)
Total	3100	1623 (52%)	1477 (48%)

## Utility and discussion

### Web-based open platform construction and data sharing

To provide high-quality slides for AI-based learning, we developed a web-based open platform, termed PAIP (http://wisepaip.org), in collaboration with Prompt Technology Ltd. PAIP was developed using Ruby on Rails, and the MARIA database was selected for the operation. Considering the open access and scaling issue of large data, we implemented PAIP on AWS EC2 RDS. In total, 1781 digital images of > 1.3 TB in size have been released through the platform thus far.

All slides released in PAIP were provided as a single set of SVS and XML files, and were accessible to all researchers. Furthermore, after submitting the consent forms for the use of data (“data use agreement”, DUA), researchers receiving approval could download the data required, and were free to use it as deemed necessary except for commercial purposes under the Creative Commons License (CC BY-NC 4.0). However, to prevent reckless data leakage and use, users were managed at four levels based on their right to use the data. Permissions according to user level are shown in Table 3.

**Table 3:** User level management

User level	Right to use			
	User guide	News	Slide search	Slide download
L0 (Unregistered user)	○	○		
L1 (Email confirmed user)	○	○	○	
L2 (DUA-approved user)	○	○	○	○
L3 (Membership)	○	○	○	○

Furthermore, L2 users (DUA-approved) were requested to submit an annual report to regularly manage user rights and follow the status of data usage, thus facilitating renewal L2 users to access the data continuously for a certain period.

**Fig. 5:**
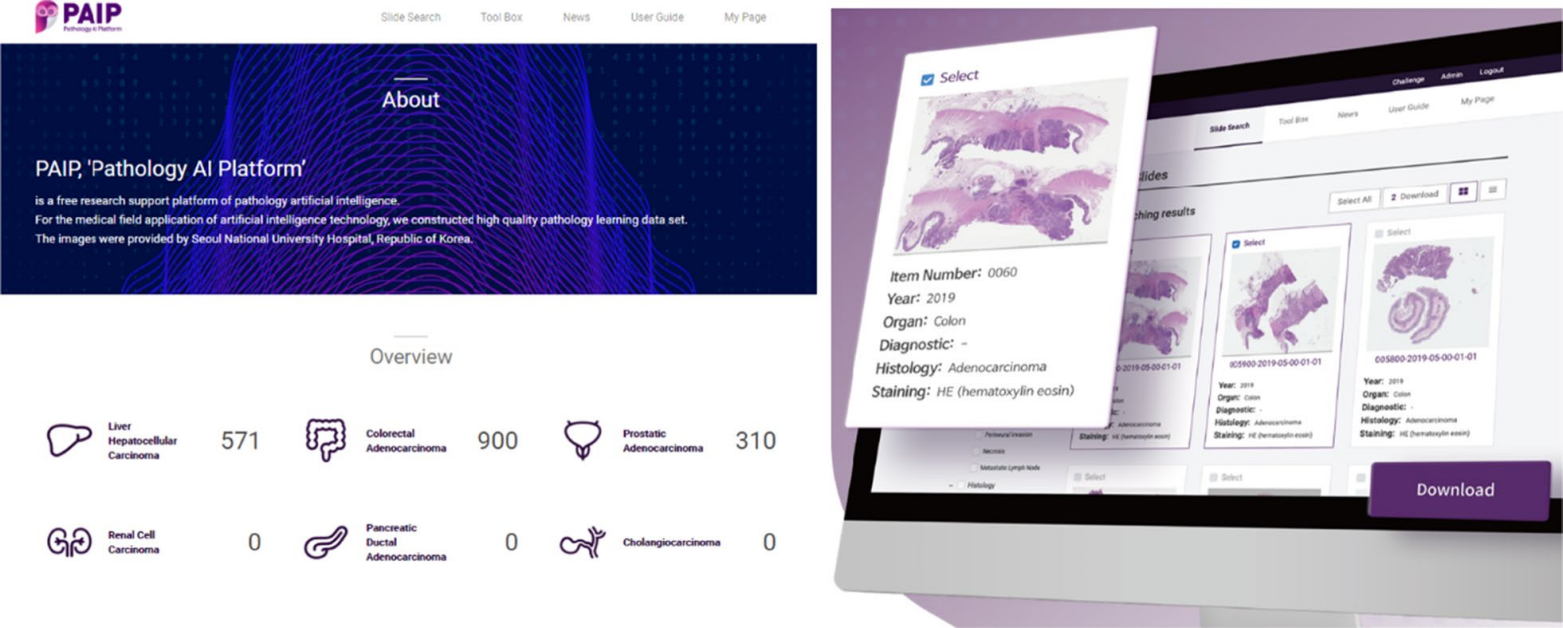
Screenshot of the pathology artificial intelligence platform and the thumbnail view

PAIP includes some visual functions to activate data utilization and enhance user convenience, while managing the user rights. All data include file names in accordance with standardized nomenclature, allowing users to select and download only the characteristics of the data that they selected with the diagnostic information filters provided by the platform. For example, if users wanted to download only IHC-stained data among colon cancer images with the adenocarcinoma histological type, they only needed to click the corresponding checkbox in the filter area. Each of the data files were arranged in the thumbnail layout, allowing users to intuitively browse and select the medical image data, and the administrator could systematically and conveniently manage the slide images (Fig. 5).

Moreover, five common pre-processing algorithms for AI modeling learners were provided on the platform. These algorithms made it easy to load and match massive images into the deep learning model at the preliminary stage. For example, one algorithm could divide the WSI into standardized patches or could connect the prediction results of the patch units to WSI units. Table 4 provides a list of the pre-processing algorithms used for this purpose.

**Table 4:** List of five pre-processing algorithms

Toolbox no	Major function
Toolbox #1	Generating and classifying patches
Toolbox #2	Smooth stitching of prediction patches
Toolbox #3	Generating heat map and overlay pyramid zoom image
Toolbox #4	SVS load and tissue mask segmentation
Toolbox #5	XML load and convert to masks

### Organization of the AI challenge

Based on the data-sharing platform established in this study, the international pathology AI challenge, PAIP2019 (https://paip2019.grand-challenge.org) was held in conjunction with MICCAI, which was deemed the “AI World Cup” of the medical community. The challenge was conducted for about 6 months with the theme of liver segmentation, and provided 100 WSIs of liver hepatocellular carcinoma from PAIP as training and test datasets. In total, 28 teams from 12 countries participated in the challenge, and participants could develop AI models using PAIP data.

## Results

Since 2018, we have been participating in government-led research projects to construct an open platform for AI learning, and have curated 3100 images by digitally imaging the pathological slides of 5 organ carcinomas to date. The data included de-identified patient information, fundamentally blocked through multiple stages of de-identification, along with annotations from skilled pathologists. The dataset thus curated was released sequentially on PAIP and will be released toward the end of 2020. Furthermore, this platform is based on the concept of the dataset provider, suggesting methods to standardize and refine pathological images with high-quality annotations for AI researchers, rendering it an intuitive platform for developers without medical knowledge to easily identify diagnostic data contained in the dataset.

### Principal findings

At the initial data collection stage, data collection proved to be difficult owing to protective regulations for patient information. In particular, South Korea Personal Information Protection Act is quite comprehensive; therefore, it was difficult to completely eliminate the risk of invading patient privacy. Further, after two consultations with a law firm, it became clear that the responsibilities to conform with this Act could not be avoided. Hence, it was difficult and time-consuming to conduct these studies with high confidence. Fortunately, in January 2020, some of these risks were resolved as amendments of three major data privacy laws (Personal Information Protection Act, Act on the Promotion of the Use of the Information Network and Information Protection, and Credit Information Use and Protection Act) were passed by the National Assembly of the Republic of Korea. Owing to different regulations among countries, when releasing internationally shared learning platforms, including this PAIP, it seems ideal to receive consent from patients at a preliminary stage.

In fact, pathologists did not have sufficient time to work in the field and faced numerous difficulties in generating the database of these high-quality data along with managing their clinical practice. The colon and liver cancer annotation, which was carried out in the early stages, was particularly challenging because pathologists curated > 1000 whole slides manually. Such tumors required different annotation levels depending on the characteristics of the carcinoma. Therefore, after 100% of the colorectal cancer data were manually annotated, the liver cancer annotation was jointly implemented by the AI team of the university. When the AI team first discerned the tumor area, pathologists could save time by correcting and confirming the annotation, thus increasing their efficiency. This effect is expected to be maximized with improvements in AI approaches. Even when applying AI approaches, pathologists still manually draw slides for training and facilitating learning by AI models. Such efforts and time are required to modify and provide feedback on the results of AI-based approaches, and marked improvements in working efficiency are now expected. Therefore, our future studies are aimed at sharing more diverse pathological images using this platform.

Finally, it is important to systematically set the file names for files containing diagnostic information for the effective accumulation and utilization of large amounts of data. In addition, it is recommended to secure adequate slots in advance in case additional diagnostic information (e.g., perineural invasions are to be added in the future) or considering scalability for linking with other platforms.

### Limitations

For the annotation phase we tried AI-assisted annotation, which is joint work with pathologists, but we did not noticeably reduce the workload of the pathologists. We trained AI models for five organs. Each model had to go through at least one more feedback cycle to achieve the desired outcome. The accuracy of each AI model varied depending on the cancer. We measured the accuracy of the AI annotation models using mIoU (mean Intersection over Union). This indicator shows how accurately AI algorithm can make a prediction compared to the ground truth given by pathologists. For the liver cancer model, the mIoU was 0.62 in the first round. When pathologists corrected the annotation of AI, the accuracy increased to 0.83. For prostate cancer and kidney cancer the accuracy of AI was 0.86 and 0.80 respectively. In the case of pancreatic cancer, the accuracy of AI annotation dropped to 0.66. The low accuracy appeared to come mainly came from the gland level annotation.

The server of the data-sharing platform was used by selecting the AWS. Although the AWS has the advantage of transferring large files owing to a wide bandwidth and paying as much as the capacity used, it has the typical feature of increased cost with increasing data accumulation and usage. Initially, the PAIP cost approximately USD 100; however, by 2020, more than USD 1000 was being spent monthly on the AWS. Therefore, the cost of using a cloud server was considered to be an economic burden. Thus, in the second stage, transition to a local server will be considered.

## Conclusions

This study describes the construction of a web-based medical AI data-sharing platform, through which we could reveal a high-quality pathology dataset annotated by pathologists. To this end, three hospitals collaborated to collect the data, who worked closely with university AI teams and a private solutions company to resolve various issues and to systematically manage massive medical image data. Thus, the constructed database offers a valuable research resource for AI researchers and for others. We intend to continue this effort and hope that our experience will help other researchers who wish to construct such platforms in the future.

## Data Availability

The datasets generated and analyzed during the current study are available in the PAIP platform, www.wisepaip.org/paip.

## Abbreviations

AWS: Amazon web services
DUA: Data use agreement
IHC: Immunohistochemistry
IRB: Institutional Review Board
PAIP: Pathology artificial intelligence platform
WSI: Whole slide image

## References

[1] 20th Global Open Data Now. National Information Society Agency (NIA). 2020. https://www.nia.or.kr/site/nia_kor/ex/bbs/View.do?cbIdx=27974&bcIdx=22015&parentSeq=22015. Accessed 08 Oct 2020.

[2] Kaggle dataset. Released by Hanyang University students. 2020. https://www.kaggle.com/kimjihoo/coronavirusdataset. Accessed 08 Oct 2020.

[3] Provisional Death Counts for Coronavirus Disease (COVID-19). Centers for Disease Control and Prevention. 2020. https://catalog.data.gov/dataset/provisional-death-counts-for-coronavirus-disease-covid-19. Accessed 08 Oct 2020.

[4] PubReliefHospService. Health Insurance Review & Assessment Service. 2020. https://www.data.go.kr/data/15043078/openapi.do. Accessed 08 Oct 2020.

[5] Litjens G, Kooi T, Bejnordi BE (2017). A survey on deep learning in medical image analysis. Med Image Anal.

[6] Wang D, Khosla A, Gargeya R, Irshad H, Beck AH. Deep learning for identifying metastatic breast cancer. arXiv preprint arXiv:1606.05718.

[7] Hwang EJ, Nam JG, Lim WH (2019). Deep learning for chest radiograph diagnosis in the emergency department. Radiology.

[8] Pasupa K, Tungjitnob S, Vatathanavaro S. Semi-supervised learning with deep convolutional generative adversarial networks for canine red blood cells morphology classification. Multimed Tools Appl 2020:1–18.

[9] Cho J, Lee K, Shin E, Choy G, Do S. How much data is needed to train a medical image deep learning system to achieve necessary high accuracy? arXiv preprint 2015; arXiv:1511.06348.

[10] Razzak MI, Naz S, Zaib A. Deep learning for medical image processing: Overview, challenges and the future. Classif BioApps 2018:323–50.

[11] Public data lake for COVID-19 research and development. AWS. 2020. https://aws.amazon.com/ko/covid-19-data-lake/. Accessed 08 Oct 2020.

[12] COVID-19 Open Research Dataset. Allen Institute for AI and MS. https://www.semanticscholar.org/cord19. Accessed 08 Oct 2020.

[13] COVID-19 dataset. Google. https://console.cloud.google.com/marketplace/details/bigquery-public-datasets/covid19-dataset-list. Accessed 08 Oct 2020.

[14] Prior F, Almeida J, Kathiravelu P (2020). Open access image repositories: high-quality data to enable machine learning research. Clin Radiol.

[15] Komura D, Ishikawa S (2018). Machine learning methods for histopathological image analysis. Comput Struct Biotechnol J.

[16] Nalisnik M, Amgad M, Lee S (2017). Interactive phenotyping of large-scale histology imaging data with HistomicsML. Sci Rep.

[17] Chang HY, Jung CK, Woo JI (2019). Artificial intelligence in pathology. J Pathol Transl Med.

[18] Xu Y, Li Y, Shen Z (2017). Parallel multiple instance learning for extremely large histopathology image analysis. BMC Bioinf.

[19] Hosny A, Parmar C, Quackenbush J, Schwartz LH, Aerts H (2018). Artificial intelligence in radiology. Nat Rev Cancer.

[20] PAIP2019. AI challenge at MICCAI. https://paip2019.grand-challenge.org/Home/. Accessed 08 Oct 2020.

[21] Schmitz R, Madesta F, Nielsen M, Werner R, Rösch T. Multi-scale fully convolutional neural networks for histopathology image segmentation: from nuclear aberrations to the global tissue architecture. arXiv 2019; arXiv:1909.10726. 10.1016/j.media.2021.101996 33647783

[22] Wang J, Xu Z, Pang Z-F, Huo Z, Luo J. Tumor detection for whole slide image of liver based on patch-based convolutional neural network. Multimed Tools Appl 2020:1–12.

[23] Enforcement rule of bioethics and safety act. http://www.law.go.kr/lsInfoP.do?lsiSeq=98198&urlMode=engLsInfoR&viewCls=engLsInfoR#0000. Accessed 08 Oct 2020.

[24] Guidelines for de-identification of personal data. https://www.kisa.or.kr/public/laws/laws2_View.jsp?mode=view&p_No=282&b_No=282&d_No=3. Accessed 08 Oct 2020.

